# MicroRNAs Discriminate Familial from Sporadic Non-BRCA1/2 Breast Carcinoma Arising in Patients ≤35 Years

**DOI:** 10.1371/journal.pone.0101656

**Published:** 2014-07-09

**Authors:** Elen Pereira Bastos, Helena Brentani, Fatima Solange Pasini, Aderbal Ruy T. Silva, Cesar Henrique Torres, Renato David Puga, Eloisa Helena Ribeiro Olivieri, Amanda Rusiska Piovezani, Carlos Alberto de Bragança Pereira, Ariane Machado-Lima, Dirce Maria Carraro, Maria Mitzi Brentani

**Affiliations:** 1 Oncology and Radiology Department, Laboratory of Medical Investigation 24 (LIM 24), University of São Paulo, Medical School, São Paulo, Brazil; 2 Laboratory of Clinical Pathology – Laboratory of Medical Investigation 23 (LIM 23), Institute and Department of Psychiatry, University of São Paulo, Medical School, São Paulo, Brazil; 3 Clinical Research Center - Hospital Israelita Albert Einstein- HIAE, São Paulo, Brazil.; 4 Laboratory of Genomics and Molecular Biology, Research Center (CIPE), A.C. Camargo Cancer Center, São Paulo, Brazil; 5 Mathematics and Statistics Institute, University of São Paulo, São Paulo, Brazil; 6 School of Arts, Sciences and Humanities, University of São Paulo, São Paulo, Brazil; University of Dundee, United Kingdom

## Abstract

The influence of genetic factors may contribute to the poor prognosis of breast cancer (BC) at a very young age. However *BRCA1/2* mutations could not explain the majority of cases arising in these patients. MicroRNAs (miRs) have been implicated in biological processes associated with BC. Therefore, we investigated differences in miRs expression between tumors from young patients (≤35 years) with sporadic or familial history and non-carriers of *BRCA1/2* mutations. Thirty-six young Brazilian patients were divided into 2 groups: sporadic (NF-BC) or familial breast cancer (F-BC). Most of the samples were classified as luminal A and B and the frequency of subtypes did not differ between familial or sporadic cases. Using real time qPCR and discriminant function analysis, we identified 9 miRs whose expression levels rather than miR identity can discriminate between both patient groups. Candidate predicted targets were determined by combining results from miRWalk algorithms with mRNA expression profiles (n = 91 differently expressed genes). MiR/mRNA integrated analysis identified 91 candidate genes showing positive or negative correlation to at least 1 of the 9 miRs. Co-expression analysis of these genes with 9 miRs indicated that 49 differentially co-expressed miR-gene interactions changes in F-BC tumors as compared to those of NF-BC tumors. Out of 49, 17 (34.6%) of predicted miR-gene interactions showed an inverse correlation suggesting that miRs act as post-transcriptional regulators, whereas 14 (28.6%) miR-gene pairs tended to be co-expressed in the same direction indicating that the effects exerted by these miRs pointed to a complex level of target regulation. The remaining 18 pairs were not predicted by our criteria suggesting involvement of other regulators. MiR–mRNA co-expression analysis allowed us to identify changes in the miR-mRNA regulation that were able to distinguish tumors from familial and sporadic young BC patients non-carriers of BRCA mutations.

## Introduction

Breast Cancer (BC) in patients below the age of 35 years is uncommon, occurring in only 2%–10% of the cases. However, this frequency may differ among different ethnic groups [1]–[6].

Despite intense treatment, the prognosis in young BC patients, particularly in black women, is worse than that in their older counterparts [2], [5], [7], [8]. This fact has been partially attributed to the high frequency of unfavorable tumor characteristics [9]–[12].

The influence of genetic factors may contribute to the poor prognosis, but familial history of cancer explains only 10%–37% of the cases, of which 10%–25% were attributable to *BRCA1/2* mutations, which are currently known as the 2 major BC predisposing genes [13], [14]. In sporadic cases, this frequency is still smaller, ranging from 3%–10% [15].

Anders and coworkers [9] have suggested that BC in young woman is a unique biological entity. Other studies have shown that its aggressive nature may be explained by the high frequency of aggressive intrinsic BC subtypes and grades, both of which were correlated with age [16], [17]. However, a recent gene expression profiling meta-analysis proposed that BC at young age appears to be biologically distinct beyond subtype distribution [18].

We recently reported a study of 54 young Brazilian patients (≤35 years). Of these, 29% presented a familial cancer history and specifically, 37.5% were carriers of germ line mutation in the *BRCA1/2* genes, which was displayed by only 8.6% of the tumors from non-familial BC cases. In addition, gene expression profiling appropriately discriminated tumors according to the presence/absence of *BRCA1/2* germ line mutations [19]. However, gene expression profile differences between familial and sporadic early onset BC patients who were not carriers of *BRCA1/2* mutations were not found.

An additional improvement of gene signatures could be found from the examination of microRNAs (miRs), which have recently emerged as important players in BC development, progression, and metastasis [20], [21]. MiRs are a class of small non-coding RNAs that post transcriptionally regulate the expression of protein-coding genes opening a new area of marker research complementary to the transcriptional gene signature. Differentially expressed miRs were identified according to different BC molecular subtypes [22], metastasis, and overall survival [23], [24]. However, little is still known about the involvement of miRs in the molecular mechanisms underlying the aggressiveness of BC in young women. An association between miR-146a phenotype and tumor age-of-onset in *BRCA1/2*-negative familial BC cases has been reported [25], [26]. In addition, a recent study highlighted that non-BRCA1/2 hereditary BC may be sub-classified using specific miR signatures [27]. Recently, Estal and coworkers suggested that the miR expression profile may facilitate the identification of sporadic BC carrying genetic/epigenetic changes in BRCA genes [28]. Our specific aims in the current study were (A) to identify a miR expression signature that could discriminate between familial and sporadic BC in young patients (≤35 years) who are non-carriers of BRCA1/2 mutations; and (B) to identify candidate target-genes related with the differentially expressed miRs.

## Materials and Methods

### Patients

Tumor samples were collected, processed and provided by A. C. Camargo Biobank (São Paulo, SP, Brazil) from breast cancer patients aged thirty five years or less, undergoing surgery, after genetic counseling and signature of an informed consent form. This study was approved by the Ethical Board for Research Project Analysis (CEP) of the A. C. Camargo Cancer Center (research protocol 1656/12) and was conducted in accordance to the Helsinki Declaration.

The BRCA1/2 status was determined in DNA extracted from peripheral blood samples, and patients with relevant mutations were excluded from subsequent analyses. Thirty-six patients who were not carriers of *BRCA1/2*, *TP53*, or *CHEK2* mutations were considered for analysis in the present study and segregated into 2 groups: familial breast cancer (F-BC) (n = 10) and sporadic breast cancer [non-familial (NF-BC)] (n = 26), according to *National Comprehensive Cancer Network* (NCCN) guidelines updated in 2011. The thirty-six patients included in the present study were also present in a previous report of our group [19]. In **Table 1**, we have reported the clinical descriptors of all these patients, including pathological features of the tumor specimens obtained from them, e.g., histological type, disease staging (TNM) at diagnosis, and histological grade. Estrogen receptor (ER), progesterone receptor (PR), and Her-2 status were determined by immunohistochemistry. Only tumor samples with distinct nuclear immunostaining in ≥10% of the cells were recorded as ER or PR-positive. Her-2 status was considered positive if the membrane staining reaction was defined as 3+. In unsure cases (2+), fluorescence *in situ* hybridization (FISH) was additionally performed. Tumors were further classified as luminal A or B, Her-2-rich, and triple negative. The classification of BC subtypes was determined by proxies of the molecular subtypes following the model proposed by Carey and coworkers [29] in which luminal A indicated ER^+^, and/or PR^+^ and HER-2^−^; subtype luminal B exhibited ER^+^, and/or PR^+^ and HER-2^+^; Her-2-rich presented Her-2 over expressed or amplified and absent ER/PR and subtype triple negative indicated ER^−^, PR^−^, HER-2^−^.

**Table 1:** Patient and sample characteristics.

ID	Age	Grade	TNM	Subtype	Familial (*NCCN criteria*)	ER	PR	Her-2
NF-BC 1	33	GN3/GH3	**IIA**	Luminal B	(-)	POS	POS	POS
NF-BC 2	35	GN3/GH2	**IIA**	Luminal B	(-)	POS	POS	POS **
NF-BC 3	33	GN3/GH3	**IIB**	Luminal A	(-)	POS	POS	NEG
NF-BC 4	25	GN2/GH2	**IV**	Trip. NEG	(-)	NEG	NEG	NEG
NF-BC 5	33	GH2/GN3	**IIB**	Luminal A	(-)	POS	NEG	NEG
NF-BC 6	30	GN2/GH2	**IIB**	Luminal B	(-)	POS	POS	POS
NF-BC 7	26	GN3/GH3	**IIB**	Trip. NEG	(-)	NEG	NEG	NEG
NF-BC 8	29	GN3/GH3	**IIA**	Luminal A	(-)	POS	NEG	NEG
NF-BC 9	33	GN2/GH2	**IIIA**	Luminal A	(-)	POS	NEG	NEG
NF-BC 10	35	GN3/GH3	**IIA**	Trip. NEG	(-)	NEG	NEG	NEG
NF-BC 11	34	GN2/GH1	**IIA**	Luminal B	(-)	POS	NEG	POS
NF-BC 12	36	GN3/GH3	**IIA**	Luminal B	(-)	POS	POS	POS
NF-BC 13	35	GN3/GH2	**IIB**	Luminal A	(-)	POS	POS	NEG
NF-BC 14	32	GH2	**IIA**	Luminal A	(-)	POS	POS	NEG
NF-BC 15	34	GN3/GH2	**IIB**	Trip. NEG	(-)	NEG	NEG	NEG
NF-BC 16	30	GH2	**IIIB**	Luminal A	(-)	POS	POS	NEG
NF-BC 17	29	GH2	**IIA**	Luminal B	(-)	POS	POS	POS
NF-BC 18	31	GH2	**IIIA**	Luminal A	(-)	POS	POS	NEG
NF-BC 19	33	GN2/GH1	**IIA**	Luminal A	(-)	POS	POS	NEG
NF-BC 20	35	GH2	**IIIB**	Luminal A	(-)	POS	POS	NEG
NF-BC 21	28	GN2/GH2	**IIA**	Luminal A	(-)	POS	POS	NEG
NF-BC 22	33	GH2/GN2	**IIB**	Luminal A	(-)	POS	NEG	NEG
NF-BC 23	33	NA	**IIA**	Luminal A	(-)	POS	NEG	NEG
NF-BC 24	26	GN3/GH2	**IIIA**	Luminal B	(-)	POS	POS	POS
NF-BC 25	22	GN3 GH2	**IIA**	Her-2	(-)	NEG	NEG	POS
NF-BC 26	34	GH2	**IIIA**	Luminal A	(-)	POS	POS	NEG
F-BC 1	28	GN3/GH2	**IIB**	Luminal B	(+)	POS	POS	POS
F-BC 2	34	GN3/GH2	**IIB**	Luminal A	(+)	POS	POS	NEG
F-BC 3	35	GN3/GH2	**IIA**	Luminal A	(+)	POS	POS	NEG
F-BC 4	29	GN3/GH3	**IIIA**	Luminal A	(+)	POS	POS	NEG
F-BC 5	29	GN3/GH3	**IIA**	Trip. NEG	(+)	NEG	NEG	NEG
F-BC 6	29	GN3/GH3	**IIB**	Luminal A	(+)	POS	POS	NEG
F-BC 7	34	GN3/GH2	**IIB**	Luminal A	(+)	POS	POS	NEG
F-BC 8	33	NA	**I**	Luminal A	(+)	POS	NEG	NEG
F-BC 9	29	NA	**I**	Luminal A	(+)	POS	POS	NEG
F-BC 10	28	GN3/GH3	**IIIB**	TripNeg	(+)	NEG	NEG	NEG

POS = positive; NEG: negative; Trip. NEG: triple negative; GN: nuclear grade; GH: histological grade; ER: estrogen receptor; PR: progesterone receptor; HER-2: growth factor receptor type 2; NCCN: *National Comprehensive Cancer Network*. ER, PR, and Her-2 receptor status was defined according to IHC.TNM =  tumor classification based on stage according to TNM criteria suggested by WHO (World Health Organization): I (T1N0M0); IIA (T0N1M0, T1N1M0, T2N0M0); IIB (T2N1M0,T3N0M0); IIIA (T0N2M0, T1N2M0, T2N2M0, T3N1M0, T3N2M0); IIIB (T4N0M0,T4N1M0, T4N2M0); IV (every T, N, and M1).

### Total RNA and DNA isolation

Frozen tumor tissue (approximately 30 mg) was homogenized with Precellys 24 equipment (Carlsbad, California, USA). The supernatant was used to purify total RNA with the RNeasy Mini kit (Qiagen, Venlo, the Netherlands) according to the manufacturer's protocol. RNA quality and concentration were assessed using a ND-1000 NanoDrop (Thermo Scientific, Wilmington, Delaware, USA) and the integrity was determined using an Agilent Bioanalyzer 2100 (Agilent Technologies, Palo Alto, California, USA). The DNA extractions from peripheral blood methodology as well as the sequencing reaction and mutation analyses were described in a recent publication of our group [19].

### MicroRNA expression profiling

A global profiling of miR expression in these 36 tumor samples was performed using the TaqMan Low Density Array Human microRNA assay panel A (TLDA, Life Technologies). The array panel A contains 377 *homo sapiens* miRs and 7 endogenous controls (ribosomal RNAs) for a total of 384 probes. Reverse transcription was performed with the RT-miRs kit and the pre-amplification with the pre-amplification kit (Life Technologies) from 350 ng of total RNA using the manufacturer's protocols. Real time PCR (RT-PCR) was performed according to the 7900 HT Real Time PCR Systems protocol using 2× Universal PCR Master Mix, no AmpErase UNG (Life Technologies).

The expression value, measured as cycle threshold (CT), of each miRs was obtained using SDS 1.2 software (Life Technologies: TaqMan OpenArray Real-Time PCR Plates). MiRs presenting expression levels below the detection limit (>38) in more than 60% of the samples were excluded from analyses. To calculate the expression of miRs for each tumor sample, the delta CT method was used and normalization was performed with the RNU48 endogenous control assay (CT of miR - CT of RNU48). The differences in miRs expression levels (fold change) were calculated from the ratio of mean 2^−ΔCT^ of a tumor sample from F-BC group relative to the mean 2^−ΔCT^ of NF-BC tumors samples [30]. The normalization between samples was performed by *limma library R version 2.13* [31].

Statistical comparisons of miRs expression between F-BC and NF-BC were performed using *Significance Analysis of Microarrays* (*SAM*) test with adjusted FDR (0%) by *MeV* program (*Multi Experiment Viewer* v.*4.5*).

### Messenger-RNA expression profiling

All 36 tumors from F-BC and NF-BC patients were included in the messenger RNA (mRNA) expression analyses. The mRNA expression profiling was performed with one-color labeled cDNAs from 500 ng of total RNA and was reverse-transcribed into double-stranded cDNA with the MMLV reverse transcriptase enzyme and primed with the oligo-dT-T7 polymerase promoter sequence. The Cy3-labeled cDNA was then transcribed *in vitro* by T7 RNA polymerase. The quantity and efficiency of the labeled amplified Cy3-cDNA were determined with a NanoDrop ND-1000 (Thermo Scientific, Wilmington, Delaware, USA). Labeled cDNAs were hybridized to the Agilent B4X44K G4112F whole human genome oligoarray (Agilent, Santa Clara, USA). All microarray raw data have been deposited in the GEO public database (http://www.ncbi.nlm.nih.gov/geo), a MIAME compliant database, under accession number GSE37126. **Table S1** shows corresponding array slide numbers to the sample ID used in the present study. Gene expression profiling was performed with a permuted student's T-test (MEV, TM4 software) using the *MeV* program (*Multi Experiment Viewer* v.*4.5*) [32], [33].

### Discriminant analyses

Linear discriminant analysis was performed to assess the ability of miRs to correctly classify patients into groups (F-BC and NF-BC). A second discriminant analysis called cross-validation [34] was performed and the proportion of samples classified on each group was recalculated.

### Targets prediction and selection of candidates

Putative targets were inferred for each miR using the miRWalk prediction program database algorithms which extract predictions from TargetScan, Diana microT 4.0, Miranda, RN22 and Pictar (http://www.umm.uni-heidelberg.de/apps/zmf/mirwalk/index.html). The final miR-target prediction results were a combination of the queries. Targeting criteria were as follows (a) near-perfect complementarity in the 7–8 nt region close to 5′-end of the miR (seed sequence) with the 3′-UTR region of target sequence; (b) conserved target sequence sites between species; (c) strong thermodynamic stability of miR–mRNA duplex; (d) complementarity between multiple sites; (e) existence of a central non-matched region (loop).

The final selection of target candidates was established by combining genes predicted by the miRWalk data base and also exhibiting differential expression from the microarray experiment profile.

### Co-expression analyses

We performed a co-expression analysis based on a method analogous to that previously described [35]. First, differentially expressed miRs and mRNAs between F-BC and NF-BC BC groups were selected and then, the co-expression correlation between each miR–mRNA interaction was calculated separately for each group using the Pearson correlation coefficient (PCC). Then, co-expression matrixes between differentially expressed genes and miRs were constructed for the F-BC and NF-BC groups. Second, to determine alterations in the co-expression pattern between the 2 groups, the absolute value of the difference of correlations in these PCCs was calculated. To determine whether the deviation in correlation between the 2 groups was significant, we randomly reassigned the patients to one of the groups and repeated the analysis. This was performed 100,000 times to calculate the random distribution. Real PCC differences for miR-RNA pairs between patient groups were compared to the random distribution to generate p values.

### Validation of miRs and targets

MiRs validation was performed by reverse transcription (RT) and quantitative PCR (qPCR) with individual TaqMan Assays from Life Technologies. From 10 ng of total RNA we synthesized cDNA using TaqMan RT reaction components following manufacture's protocol (Life Technologies), and qPCR was performed in duplicates and accordantly to the 7900 HT Real Time PCR Systems protocol using 2× Universal PCR Master Mix, no Amp Erase UNG (Life Technology).

For mRNA-target validation the RT was performed with Super Script III – First Strand Synthesis Super Mix (Invitrogen Life Technologies) using random hexamer primers (0.05 µg/µL) and total RNA (1 ug). The cDNA samples (2 ng) were subjected to qPCR assays in triplicate using SYBR Green methodology with Power SYBR Green PCR Master Mix (Applied Biosystems, Life Technologies), followed by 7900 Real Time (Life Technologies). Gene-specific primers were designed using the Primer 3 software (http://frodo.wi.mit.edu/primer3/) to generate a PCR product in the 3′ portion, spanning the translated region of the target mRNA. Sequences present in different exons, preferentially separated by long introns, were selected, according with sequences deposited at http://www.ncbi.nlm.nih.gov/nucleotide. To avoid non-specific product formation, BLAST analysis (www.ncbi.nlm.nih.gov/blast) was carried out. The reaction conditions used to all primers were: 95°C for 10 minutes, followed by 40 cycles at 95°C for 15 seconds and annealing temperature of 59°C,60°C or 62°C for 60 seconds (**Table S2**).

To calculate the expression of miRs for each tumor sample, the delta delta CT method was used and normalization was performed with the RNU48 endogenous. The F-BC 11sample was considered as reference.

To calculate the expression of target-mRNAs, NF-BC 33 sample was considered as reference sample based on Pfaffl method: ratio = (E_target_)^ΔCP target (ACTB–sample)^/(E_NF-BC 33_) ^ΔCP NF-BC 33 (ACTB–sample)^). The differences in miRs and target expression levels (fold change) were calculated from the ratio of 2^−ΔΔCT^ or Pfaffl ratio, respectively, of F-BC group relative to NF-BC tumor samples.

## Results

All 36 tumors were classified as infiltrative ductal carcinomas and displayed grade 2 or 3. The clinical stage at diagnosis as well as the histological grade and tumor markers were similarly distributed in sporadic or familial cases. Most of them were classified as luminal A and B and the frequency of the subtypes did not differ between familial or sporadic cases (**Table 2**).

**Table 2:** Frequency of clinical and histopathological characteristics according to the groups—familial breast cancer (F-BC) and sporadic breast cancer (NF-BC).

Clinic status	Category	F-BC n (%)	NF-BC n (%)	TOTAL n (%)	P
**Stage**	I-II	8 (80)	19 (73)	27 (75)	1.0
	III-IV	2 (20)	7 (27)	9 (25)	
**Histological grade**	I-II	4 (50)	18 (75)	22 (68.8)	0.38
	III	4 (50)	6 (25)	10 (31.2)	
**Nuclear grade**	II	0 (0)	6 (33.3)	6 (16.6)	0.13
	III	8 (100)	12 (66.7)	20 (55.5)	
**ER**	Positive	7 (70)	21 (81)	28 (78)	0.64
	Negative	2 (20)	6 (23)	8 (22)	
**PR**	Positive	7 (70)	15 (58)	22 (61)	0.70
	Negative	3 (30)	11 (42)	14 (39)	
**Her-2**	Positive	1 (10)	8 (31)	9 (25)	0.39
	Negative	9 (90)	18 (69)	27 (75)	

### Differentially expressed miRs

In an attempt to identify a miR signature that separates familial and sporadic breast carcinoma, we profiled miR expression on 36 tumor samples derived from 26 sporadic (NF-BC) and 10 familial patients (F-BC), using a miR platform containing 377 miRs. As 1 sample (F-BC 3 in **Table 1**) was excluded from the analyses due to a weak normalization result, only 35 tumor samples were further analyzed.

Among the 377 miRs contained in the array panel, 121 showed an expression level below the detection limit (CT >38) in more than 60% of the samples in both groups and were excluded for downstream analyses. While comparing F-BC and NF-BC using a Significance Analysis of Microarrays (SAM) statistical test (FDR = 0 and a delta value of 0.77), 9 miRs out of 256 showed significantly differentiated expression. Among them, 3 miRs (miR-486-3p, miR-874, and miR-98) were downregulated, whereas 6 miRs (miR-124, miR-210, miR-381, miR-455-3p, miR-501-5p, and miR-660) were upregulated in F-BC tumors as compared with tumors of NF-BC patients (**Table 3**).

**Table 3:** MiRs differentially expressed between F-BC and NF-BC.

miRs	Fold-change (F-BC/NF-BC)
hsa-miR-124	10.08
hsa-miR-210	7.32
hsa-miR-455-3p	4.28
hsa-miR-660	2.65
hsa-miR-381	2.47
hsa-miR-501-5p	2.15
hsa-miR-98	−2.29
hsa-miR-486-3p	−4.53
hsa-miR-874	−4.70

Fold change expression between comparison of F-BC and NF-BC was considered significant with an FDR (false discovery rate) = 0 and a delta value of 0.77.

### Discriminant analyses

**Figure 1:**
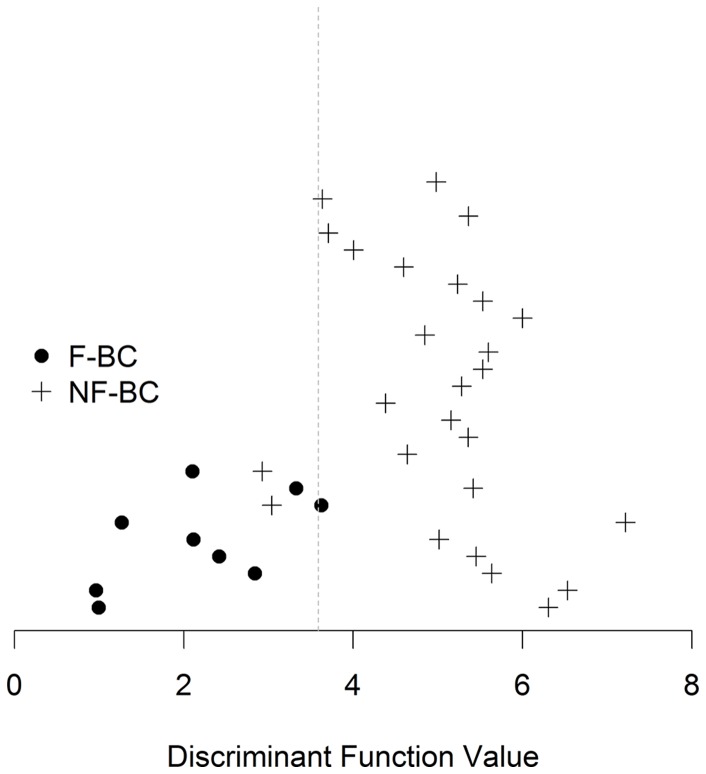
Cross-validation analysis graphic. Representative *cross-validation analysis* graphic of 35 patients from the F-BC and NF-BC groups. Black spots indicate F-BC samples while plus signs indicate NF-BC samples. The line represents the limit discriminant function between groups. F-BC, familial breast cancer; NF-BC, non-familial breast cancer.

A discriminant analyses indicated that the 9 miRs differentially expressed could discriminate tumors between F-BC and NF-BC. The graphic (**Figure 1**) indicated 82% of accuracy in the distribution of the 35 patients between both groups. Out of 35 patients, 3 (NF-BC 7, NF-BC 9, and F-BC 8) were not correctly classified.

On cross-validation analysis, 1 patient was removed from the analysis, a new discriminant function was estimated and the removed patient was reclassified. Performing this procedure with all the patients, we had a classification accuracy of 88% and 92% for F-BC and NF-BC patients, respectively.

### Differentially expressed mRNA

Differences in gene expression profiling of F-BC compared to NF-BC were assessed with a permuted student's T-test and genes were considered differentially expressed when P≤0.01. The gene expression profile led to 1599 probes representing 1415 unique differentially expressed genes. Among 1415, 742 probes were downregulated and 857 probes were upregulated.

### Targeting prediction and selection of candidate genes

**Figure 2:**
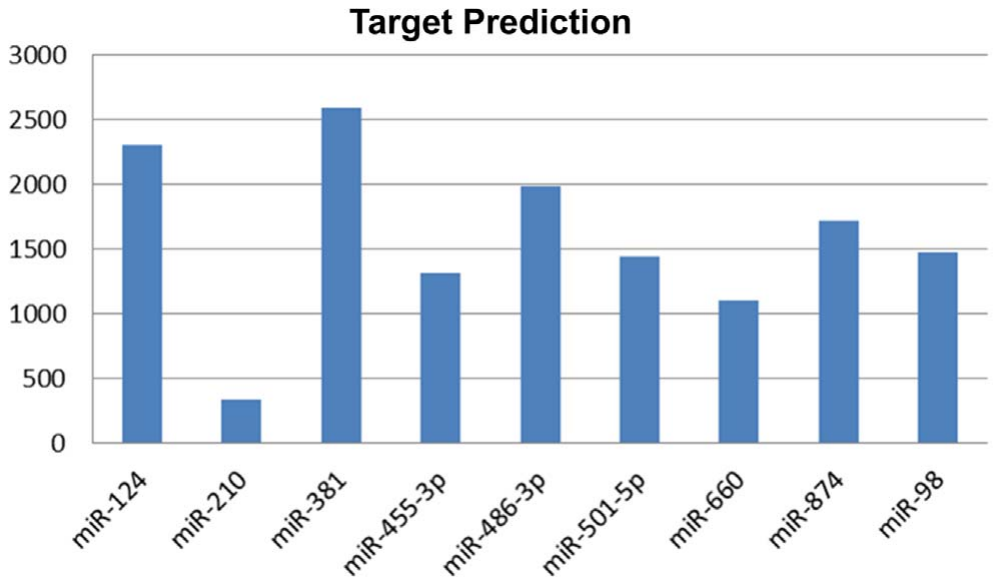
Target prediction graph. Graph displaying the number of predicted targets from miRWalk for each miRNA differentially expressed between F-BC and NF-BC groups. F-BC, familial breast cancer; NF-BC, non-familial breast cancer.

The number of predicted targets for each of the 9 differentially expressed miRs among F-BC and NF-BC patients, ranged from 2594 unique targets for miR-381 to 342 targets for miR-210, for a total of 14294 targets (**Figure 2**). To select candidate genes, we combined the list of 1415 differentially expressed genes from our microarray experiment with the 14294 predicted targets from the miRWalk data base and examined only the intersection. This analysis yielded 91 unique differentially expressed predicted target-genes correlated with at least 1 out of 9 miR regulators. Out of 91, 34 showed down expression and 57 showed over expression. Supposedly candidate target-gene should exhibit an inverse expression level compared to the correspondent miR, however we also found miRs that were coordinately correlated with their targets. (**Table S3**).

### Co-expression analyses

**Figure 3:**
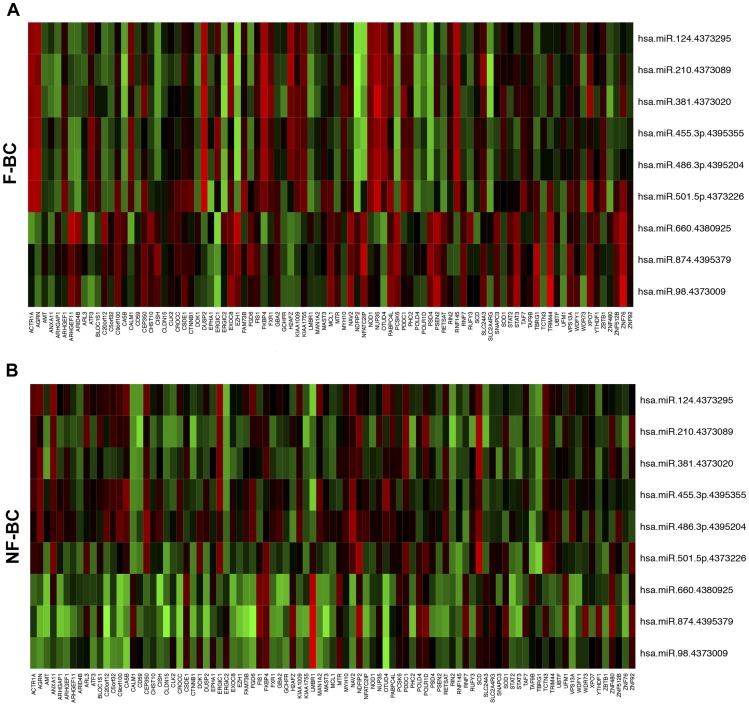
Co-expression matrixes. Co-expression matrixes of the 91 differentially expressed genes vs 9 differentially expressed miRs for F-BC (A) and NF-BC (B), respectively. The colors represent the co-expression values reaching from 1 to −1 for red and green, respectively. F-BC, familial breast cancer; NF-BC, non-familial breast cancer.

**Figure 4:**
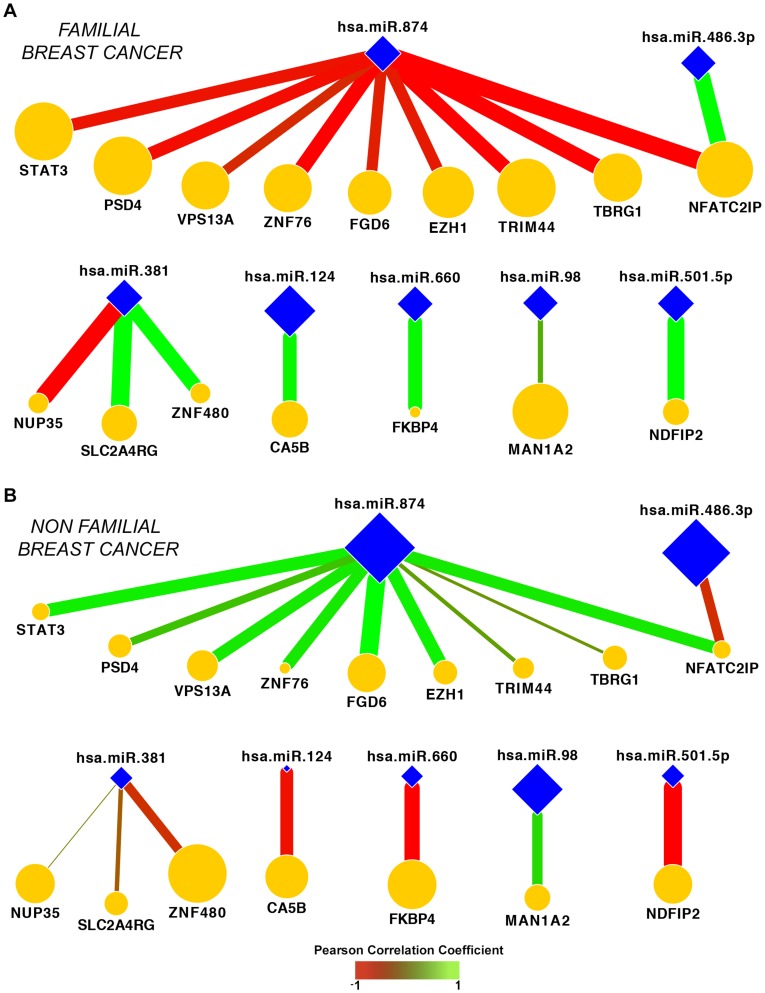
Illustration of network signatures. Seventeen miR–mRNA predicted interactions whose co-expression are significantly different between F-BC (A) and NF-BC (B) groups. Color edges represent positive (green) or negative (red) Pearson correlation values. The edge thickness indicates the magnitude of Pearson correlation values. The node size is proportional to the fold change of genes (orange nodes) and of miRs (blue nodes) between F-BC to NF-BC groups. F-BC, familial breast cancer; NF-BC, non-familial breast cancer.

To assess the differences of miRs and gene interaction patterns between F-BC and NF-BC, we performed co-expression analysis. Co-expression matrixes with 91 candidate genes against 9 miRs were performed separately for the F-BC and NF-BC groups (**Figures 3A and 3B**). We found 49 miR–mRNA interactions, including those with predicted or not predicted genes for correspondent miR, which presented statistically significantly differences in co-expression between F-BC and NF-BC (P<0.05) (**Table S4**). Not predicted interactions were defined as those showing differences in co-expression between the groups but in which miR-mRNA interactions not fulfilled our target prediction criteria. Once miRs have a negative regulatory role on their mRNA targets, we selected miRs–mRNA interaction fulfilling the condition that the expression levels of the genes should be inversely correlated with their corresponding miRs. In other words, if a given miR was upregulated, the expression of its target is expected to be downregulated and vice-versa. From the 31 predicted miR–mRNA interactions, 17 pairs presented inverse fold-change values between F-BC and NF-BC. These results suggested that 17 predicted miR–mRNA interactions could be supported by the potential miRs post-transcription regulator function. Analysis of those miR–mRNA interactions defined a network of 16 genes and 7 miRs whose co-expression is different in F-BC and NF-BC (**Figures 4A and 4B**). Comparing both network profiles, F-BC against NF-BC, we observed different colors of edges representing negative (red) or positive (green) co-expression correlation as well as the different thickness of the edges, where thicker edges indicate high values of co-expression correlation, and thinner edges represent low values of co-expression correlation. We can also visualize that 11 genes from the NF-BC group exhibited downregulation (smaller orange nodes) and 5 genes upregulation (larger orange nodes) compared with the same genes in the F-BC group. On the other hand, we observed 7 miRs (blue nodes) of which miR-98, miR-486-3p, and miR-874 showed low values of co-expression correlation (smaller size of blue nodes) and miR-124, miR-381, miR-501-5p, and miR-660 showed high values of co-expression correlation (bigger size of blue nodes) in F-BC compared with NF-BC network profile.

Out of 49, 8 not predicted miR–mRNA interactions showing inverse correlation (**Table S5**) could also distinguish F-BC from NF-BC tumors. Among them 5 pairs could be considered as predicted targets albeit using less stringent criteria.

From 49 miR–mRNA interactions, 24 (14 predicted and 10 not predicted) showed differentially expressed genes and significant co-expression differences; however, they did not exhibit inverse signal of fold-change value, suggesting that the separation between F-BC from NF-BC tumors could not be exclusively explained by the predominant mechanism of miRs-mediated gene repression.

We performed functional analysis using the IPA (Ingenuity Pathway Analysis; QUIAGEN) program on a set formed by 28 unique genes representing 49 predicted/not predicted miR-mRNA interactions listed in **table S4**. The results from functional analysis demonstrated over representation of some biological processes involved with: apoptosis, cell death, and fibroblast proliferation (**Table S6**).

### Validation of miRs and targets

The miRs and target-genes validation experiments were performed in a panel of independent samples of sporadic BC (n = 9), with low risk of displaying BRCA1/2 mutation (<12%) according to *Breast and Ovarian Analysis of Disease Incidence and Carrier Estimation Algorithm* (BOADICEA) (http://ccge.medschl.cam.ac.uk/boadicea/). Due to difficulties in acquisition of new cases of fresh frozen tissues of familial BC cases of young patients, not harboring BRCA1/2 mutation, the set of validation included only 2 new samples totalizing 7 samples of F-BC group. All validation set of patients exhibited tumors with luminal subtype. Other clinical and histopathological characteristics were reported in **table S7**.

All 9 miRs were analyzed but the results of qPCR experiments showed the expected upregulation only for miR-124, miR-210, miR-381, miR-455-3p, miR-501-5p, miR-660 and miR-874 down regulation in the F-BC group versus the NF-BC group in accordance to the main results. The small number of samples in the validation experiments might explain the lack of miR-486 and miR-98 validation.

We selected 10 predicted target-genes (**Table S2**) from the network miR–mRNA interactions signature that showed inverse correlation, following **figure 4**, to confirm their inverse expression related to correspondent miR based on fold change values.

Out of 10, 8 target-genes exhibited inverse fold change values with their respectively miRs in agreement with our main results (**Table 4**). This remarkable validation suggested that miR-124, miR-210, miR-381, miR-455-3p, miR-501-5p, miR-660 and miR-874, as well as STAT3, PSD4, SNF480, FGD6, EZH1, TRIM44, TBRG1, NFATC2IP and CA5B target-genes could possibly explain molecular mechanisms involved in BC carcinogenesis that distinguished tumors from familial to sporadic BC in young patients BRAC1/2 non carriers mutations.

**Table 4:** MiR-mRNA interactions validation.

miR-mRNA interaction	Fold miR	Fold mRNA-target	Validated
miR-124:CA5B	87.6	−100.0	Validated
miR-381:ZNF480	10.2	1.29	Not validated
miR-98: MAN1A2	3.68	−2.38	Not validated*
miR-874:STAT3	−2.5	2.97	Validated
miR-874:PSD4	−2.5	2.05	Validated
miR-874:FGD6	−2.5	3.44	Validated
miR-874:EZH1	−2.5	3.78	Validated
miR-874:TRIM 44	−2.5	3.00	Validated
miR-874:TBRG1	−2.5	2.45	Validated
miR-874:NFATC2IP	−2.5	2.16	Validated

*miR-mRNA interaction without agreement with our main results.

## Discussion

We present the results of integrated analysis of miR/mRNA data from the same tumor tissue samples to identify genes that could differentiate between tumor harvested from young patients (aged ≤35 years) with familial BC and those from sporadic BC, not harboring BRCA1/BRCA2 mutations. We identified a set of 9 miRs whose expression levels, rather than miR identity, were able to correctly separate, with high accuracy, familial and non-familial young BC patients. A subset of these miRs has previously been characterized as BC regulatory genes, including miR-486-3p [36], miR-98 [37], miR-874 [23], miR-210 [24], miR-124 [38], [39], whereas miR-660 [40], [41] has been associated with other cancers or tissue types.

We next identified a set of miRs showing significant negative or positive correlations with those of their targets. Approximately 34,6% retained inversely correlated miR–mRNA interactions. An interaction network revealed changes in the co-expression of these miR-mRNA pairs that were able to distinguish familial from sporadic breast cancer. For instance, a decreased expression of miR-874, miR-98 and miR-486-3p were associated with increased expression of their predicted target genes in F-BC cases. MiR-874, which has been previously associated with unfavorable prognosis in invasive breast cancer [23] was inversely correlated with several of their paired genes (EZH1, FGD6, PSD4, NFAIC2IP, STAT3, TBRG1, TRIM44, VPS13A, ZNF76), suggesting that miR-874 has a critical role in the regulation of genes preferentially expressed in F-BC, compared with the sporadic cases. This analysis revealed genes involved in embryonic stem cell self-renewal, such as STAT3 and EZH1 [42]-[44]. FGD6 (annexin A2) is a mediator of EGFR endocytosis and its inhibition in BC coincided with an enhanced EGF-signaling [45]. TBRG1 (NIAM) was previously identified as one of the TGFβ1-responsive genes and has been described as a novel growth inhibitor that contributes to the maintenance of chromosomal stability [46]. VPS13A codes for vacuolar sorting proteins, and its loss was observed in colorectal and gastric cancers with high microsatellite instability [47]. Upregulation of NFATC2IP (nuclear factor of activated T-cells, cytoplasmic, calcineurin-dependent 2 interacting protein) can induce the expression of ILs that stimulates T cell proliferation and activation. TRIMM44 is a member of tripartite motif-containing protein (TRIM) family, which is an important regulator of carcinogenesis [48].

Contrariwise, upregulated miRs in familial versus sporadic cases were correlated with a reduced expression of their target genes, including CA5B (miR-124), ZNF480, SLC2A4RG, NUP35 (miR-381), NDFIP2 (miR-501-5p), and FKBP4 (miR-660). FKBP4, a binding protein of SSEA-4 is a syalyl-glycolipid that has been commonly used as a pluripotent human embryonic stem cell marker. The inhibition of FKBP4 could reduce the expression of SSEA-4, leading to suppression of cancer malignant processes [49]. SLC2A4R6 is associated with the recruitment of glut4 to the plasma membrane and its downregulation may decrease glucose uptake and AKT signaling [50]. CA5B is an enzyme localized in the mitochondrial matrix that converts the CO_2_ produced by the TCA cycle to HCO^3−^, which in turn controls metabolic pathways that increase oxidative phosphorylation. A decrease in CA5B levels may lead to a drop in intracellular pH and an activation of the pro-apoptotic protein BAX [51]. Thus, gene profiling in familial BC appears to be associated with some biological processes that seem to characterize a less aggressive behavior compared to the sporadic cases.

Our findings of coordinated expressed pairs between miR and mRNA predicted levels (28.6%) indicated that some miRs, including miR-501-5p, miR-660, miR-874, miR-98, miR-124, and miR-455-3p, act as positive regulators of their target mRNAs. The targets of this population appear to include mainly mRNAs associated with the embryonic development or the nervous system. Several instances of miR co-expression in the same direction as their target genes have been previously reported, albeit this is a less well understood phenomenon [52]–[54]. We also detected a set of genes that, although presenting positive/negative co-expression, were not included in our list of *in silico* predicted targets, indicating that these genes are not potential miR targets according to our stringent criteria, however, they may be regulated by other mechanisms.

In conclusion, comparing tumors of young patients with or without familial BC history not carriers of BRCA1/2 mutation our results showed similarity between their phenotypes, most tumors of the present series being of the luminal subtype corroborating previous results [55]. However by applying co-expression analysis we found out transcriptional differences between both groups highlighting that changes in the miR-mRNA regulation were able to distinguish tumors between both groups.

## Supporting Information

Table S1 **Microarray raw data from GEO accession number GSE37126 showing the correspondent samples ID of the present study.**
(PDF)

Table S2 **Target-genes selected to validation.**
(PDF)

Table S3 **91 differently expressed candidate genes.** List of 91 differentially expressed genes associated to at least one miR.
(PDF)

Table S4 **49 miRNA–mRNA interactions.** Interactions presenting significant differences of co-expression profile between F-BC and NF-BC.
(PDF)

Table S5 **miR–mRNA not predicted interactions, presenting inverse correlation.**
(PDF)

Table S6 **Biological processes.** Categories over represented by the target genes related to 49 miR-mRNA interactions.
(PDF)

Table S7 **Patient and tumor characteristics of the validation set.**
(PDF)
