# Supplementary material for: MicroRNAs Discriminate Familial from Sporadic Non-BRCA1/2 Breast Carcinoma Arising in Patients ≤35 Years
Source: PLoS One. 2014 Jul 9;9(7):e101656. doi: 10.1371/journal.pone.0101656 (PMC4090167; doi:10.1371/journal.pone.0101656)
Supplement: Table S1 — Microarray raw data from GEO accession number GSE37126 showing the correspondent samples ID of the present study. (PDF) [file pone.0101656.s001.pdf]

Table S1. Microarray raw data from GEO accession number GSE37126 showing the correspondent samples ID of the present study

Microarray raw from GEO accession number GSE37126	Sample ID	Sample ID in the present study
GSM911452	MJ01001T	NF-BC 1
GSM911453	MJ01004T	NF-BC 2
GSM911454	MJ01006T	NF-BC 3
GSM911455	MJ01007T	NF-BC 4
GSM911456	MJ01008T	NF-BC 22
GSM911457	MJ01009T	NF-BC 5
GSM911458	MJ01011T	F-BC 9
GSM911459	MJ01014T	F-BC 8
GSM911460	MJ01015T	NF-BC 23
GSM911461	MJ02001T	F-BC 1
GSM911462	MJ02002T	NF-BC 6
GSM911463	MJ02003T	NF-BC 7
GSM911464	MJ02004T	F-BC 2
GSM911465	MJ02005T	NF-BC 8
GSM911466	MJ02007T	F-BC 4
GSM911467	MJ02008T	NF-BC 9
GSM911468	MJ02012T	F-BC 5
GSM911469	MJ02013T	F-BC 6
GSM911470	MJ02014T	NF-BC 10
GSM911471	MJ02015T	F-BC 7
GSM911472	MJ02016T	F-BC 3
GSM911473	MJ02019T	NF-BC 11
GSM911476	MJ02024T	NF-BC 12
GSM911479	MJ02027T	NF-BC 13
GSM911480	MJ02028T	NF-BC 14
GSM911483	MJ02033T	NF-BC 15
GSM911485	MJ02036T	NF-BC 24
GSM911486	MJ02037T	F-BC 10
GSM911487	MJ02038T	NF-BC 25
GSM911492	MJ04011T	NF-BC 17
GSM911495	MJ04015T	NF-BC 18
GSM911496	MJ04019T	NF-BC 19
GSM911497	MJ04020T	NF-BC 20
GSM911498	MJ04025T	NF-BC 16
GSM911499	MJ04027T	NF-BC 26
GSM911500	MJ04028T	NF-BC 21
