# Supplementary material for: MicroRNAs Discriminate Familial from Sporadic Non-BRCA1/2 Breast Carcinoma Arising in Patients ≤35 Years
Source: PLoS One. 2014 Jul 9;9(7):e101656. doi: 10.1371/journal.pone.0101656 (PMC4090167; doi:10.1371/journal.pone.0101656)
Supplement: Table S2 — Target-genes selected to validation. (PDF) [file pone.0101656.s002.pdf]

Table S2. Target-genes selected to validation.

Gene symbol	Primer sequence (5´- 3´)	Anneling temperature	Product size (bp)
STAT3	F: AGTATTGTCGGCCAGAGAGC<br>R: GGTGTCACACAGATAAACTTGGTC	60	91
EZH1	F: TTTCATGCCACCCCTAATGT<br>R:TGGCATACTCCTTTGCTCCT	60	112
CA5B	F: GCCTCCACGTCTGGAATAAT<br>R: CCCCAGTGAAAATGGAACTG	60	127
FGD6	F: CCCAAGAAGCCTATCAGAATG<br>R: TCGCTTCTAGCCATTCATCC	59	87
NFATC2IP	F: GTGCAGGGAAAGGAGAAACA<br>R: ACAGTCCCATGGCCTCCT	62	100
MAN1A2	F: GGTGGGTTTTCTGGAGTCAA<br>R: AGGTCATCACCGGAGAACAG	60	122
PSD4	F: CCTGCAGAGACTGGAGACG<br>R: CCTTCCTCTGGGGACTGTG	60	131
ZNF480	F: ACTCCATGTTGGAGCAAAGG<br>R: TCCCAATGCATAGCTGCTC	60	122
TBRG1	F: ATCAAGGATGGTGGTGTGC<br>R: CTGGAAGCAGGTTAGGCATT	60	154
TRIM 44	F: GATGACTCAGATGGCCCAAG<br>R: AGCAAGCCTTCATGTGTCCT	60	97
