# Supplementary material for: MicroRNAs Discriminate Familial from Sporadic Non-BRCA1/2 Breast Carcinoma Arising in Patients ≤35 Years
Source: PLoS One. 2014 Jul 9;9(7):e101656. doi: 10.1371/journal.pone.0101656 (PMC4090167; doi:10.1371/journal.pone.0101656)
Supplement: Table S3 — 91 differently expressed candidate genes. List of 91 differentially expressed genes associated to at least one miR. (PDF) [file pone.0101656.s003.pdf]

Table S3. 91 differently expressed candidate genes. List of 91 differentially expressed genes associated to at least one miR.

Target-gene	Fold gene	Fold miR-124	Fold miR-210	Fold miR-381	Fold miR-455-3p	Fold miR-486-3p	Fold miR-501-5p	Fold miR-660	Fold miR-874	Fold miR-98
AGRN	2,12	10,08	NP	NP	NP	NP	NP	NP	NP	NP
ANXA11	-1,45	10,08	NP	2,47	NP	NP	NP	NP	NP	NP
ARHGAP1	1,67	10,08	NP	2,47	NP	-4,54	NP	NP	NP	NP
ARHGEF1	1,70	10,08	NP	2,47	NP	-4,54	NP	NP	NP	NP
CA5B	-1,93	10,08	NP	2,47	NP	-4,54	NP	NP	NP	NP
CD59	1,88	10,08	7,32	2,47	NP	-4,54	2,16	2,66	-4,71	-2,30
CEP350	1,35	10,08	7,32	2,47	4,29	-4,54	2,16	2,66	-4,71	-2,30
CHST10	1,84	10,08	7,32	2,47	4,29	-4,54	2,16	2,66	-4,71	-2,30
CSDE1	-1,38	10,08	7,32	2,47	4,29	-4,54	2,16	2,66	-4,71	-2,30
FXR1	1,09	10,08	7,32	2,47	4,29	-4,54	2,16	2,66	-4,71	-2,30
KIAA1755	2,09	10,08	7,32	2,47	4,29	-4,54	2,16	2,66	-4,71	-2,30
MAN1A2	1,58	10,08	7,32	2,47	4,29	-4,54	2,16	2,66	-4,71	-2,30
MTR	1,38	NP	7,32	2,47	4,29	-4,54	2,16	2,66	-4,71	-2,30
MYH10	1,74	NP	7,32	2,47	4,29	-4,54	2,16	2,66	-4,71	-2,30
NAV2	2,34	NP	7,32	2,47	4,29	-4,54	2,16	2,66	-4,71	-2,30
OTUD4	2,18	NP	7,32	2,47	4,29	-4,54	2,16	2,66	-4,71	-2,30
PABPC4L	1,62	NP	7,32	2,47	4,29	-4,54	2,16	2,66	-4,71	-2,30
PCSK6	3,00	NP	7,32	2,47	4,29	-4,54	2,16	2,66	-4,71	-2,30
PSD4	1,69	NP	7,32	2,47	4,29	-4,54	2,16	2,66	-4,71	-2,30
SCD	-1,85	NP	7,32	2,47	4,29	-4,54	2,16	2,66	-4,71	-2,30
STAT2	1,96	NP	7,32	2,47	4,29	-4,54	2,16	2,66	-4,71	-2,30
STAT3	1,81	NP	7,32	2,47	4,29	-4,54	2,16	2,66	-4,71	-2,30
TBRG1	1,31	NP	7,32	2,47	4,29	-4,54	2,16	2,66	-4,71	-2,30
UBTF	1,75	NP	7,32	2,47	4,29	-4,54	2,16	2,66	-4,71	-2,30
WDFY1	1,57	NP	7,32	2,47	4,29	-4,54	2,16	2,66	-4,71	-2,30
ACTR1A	-1,46	NP	7,32	2,47	4,29	-4,54	2,16	2,66	-4,71	-2,30
SLC2A4RG	-1,40	NP	7,32	2,47	4,29	-4,54	2,16	2,66	-4,71	-2,30
ZNF512B	1,55	NP	7,32	2,47	4,29	-4,54	2,16	2,66	-4,71	-2,30
FAM73B	1,58	NP	7,32	2,47	4,29	-4,54	2,16	2,66	-4,71	-2,30
ZNF480	-1,76	NP	NP	2,47	4,29	-4,54	2,16	2,66	-4,71	-2,30
ARID4B	1,47	NP	NP	2,47	4,29	-4,54	2,16	2,66	-4,71	-2,30

Target-gene	Fold gene	Fold miR-124	Fold miR-210	Fold miR-381	Fold miR-455-3p	Fold miR-486-3p	Fold miR-501-5p	Fold miR-660	Fold miR-874	Fold miR-98
YTHDF1	-1,38	NP	NP	2,47	4,29	-4,54	2,16	2,66	-4,71	-2,30
SLC24A3	2,07	NP	NP	2,47	4,29	-4,54	2,16	2,66	-4,71	-2,30
NUP35	-1,42	NP	NP	2,47	4,29	-4,54	2,16	2,66	-4,71	-2,30
EXOC8	1,53	NP	NP	2,47	4,29	-4,54	2,16	2,66	-4,71	-2,30
CTNNB1	-1,08	NP	NP	2,47	4,29	-4,54	2,16	2,66	-4,71	-2,30
RNF145	1,57	NP	NP	2,47	4,29	-4,54	2,16	2,66	-4,71	-2,30
ZNF92	-1,08	NP	NP	2,47	4,29	-4,54	2,16	2,66	-4,71	-2,30
KIAA1009	1,05	NP	NP	2,47	4,29	-4,54	2,16	2,66	-4,71	-2,30
XPO7	1,54	NP	NP	2,47	4,29	-4,54	2,16	2,66	-4,71	-2,30
TCTN3	-1,32	NP	NP	2,47	4,29	-4,54	2,16	2,66	-4,71	-2,30
MCL1	1,57	NP	NP	2,47	4,29	-4,54	2,16	2,66	-4,71	-2,30
RIN2	1,33	NP	NP	2,47	4,29	-4,54	2,16	2,66	-4,71	-2,30
FGD6	1,60	NP	NP	2,47	4,29	-4,54	2,16	2,66	-4,71	-2,30
PSEN2	1,33	NP	NP	2,47	4,29	-4,54	2,16	2,66	-4,71	-2,30
SOD1	-1,52	NP	NP	2,47	4,29	-4,54	2,16	2,66	-4,71	-2,30
TAF7	-1,78	NP	NP	2,47	4,29	-4,54	2,16	2,66	-4,71	-2,30
CALM1	-1,55	NP	NP	2,47	4,29	-4,54	2,16	2,66	-4,71	-2,30
NFATC2IP	1,57	NP	NP	2,47	4,29	-4,54	2,16	2,66	-4,71	-2,30
ARHGEF11	1,83	NP	NP	2,47	4,29	-4,54	2,16	2,66	-4,71	-2,30
H2AFZ	-1,75	NP	NP	NP	4,29	-4,54	2,16	2,66	-4,71	-2,30
DOK1	1,18	NP	NP	NP	4,29	-4,54	2,16	2,66	-4,71	-2,30
DUSP2	2,02	NP	NP	NP	4,29	-4,54	2,16	2,66	-4,71	-2,30
MAST3	1,60	NP	NP	NP	4,29	-4,54	2,16	2,66	-4,71	-2,30
RETSAT	1,74	NP	NP	NP	4,29	-4,54	2,16	2,66	-4,71	-2,30
C20orf12	1,64	NP	NP	NP	4,29	-4,54	2,16	2,66	-4,71	-2,30
VPS13A	1,41	NP	NP	NP	4,29	-4,54	2,16	2,66	-4,71	-2,30
CISH	2,11	NP	NP	NP	NP	-4,54	2,16	2,66	-4,71	-2,30
CLK2	1,66	NP	NP	NP	NP	-4,54	2,16	2,66	-4,71	-2,30
FKBP4	-1,21	NP	NP	NP	NP	-4,54	2,16	2,66	-4,71	-2,30
CLDN15	1,74	NP	NP	NP	NP	-4,54	2,16	2,66	-4,71	-2,30
GCHFR	-2,24	NP	NP	NP	NP	-4,54	2,16	2,66	-4,71	-2,30
PDDC1	-1,32	NP	NP	NP	NP	-4,54	2,16	2,66	-4,71	-2,30

Target-gene	Fold gene	Fold miR-124	Fold miR-210	Fold miR-381	Fold miR-455-3p	Fold miR-486-3p	Fold miR-501-5p	Fold miR-660	Fold miR-874	Fold miR-98
ARL3	-1,76	NP	NP	NP	NP	-4,54	2,16	2,66	-4,71	-2,30
ATF3	2,23	NP	NP	NP	NP	-4,54	2,16	2,66	-4,71	-2,30
FIS1	-1,38	NP	NP	NP	NP	-4,54	2,16	2,66	-4,71	-2,30
ZNF76	1,70	NP	NP	NP	NP	-4,54	2,16	2,66	-4,71	-2,30
C5orf32	-1,48	NP	NP	NP	NP	-4,54	2,16	2,66	-4,71	-2,30
EPHA1	1,76	NP	NP	NP	NP	NP	2,16	2,66	-4,71	-2,30
ERGIC2	-1,47	NP	NP	NP	NP	NP	2,16	2,66	-4,71	-2,30
NOD1	1,51	NP	NP	NP	NP	NP	2,16	2,66	-4,71	-2,30
TAF9B	-1,14	NP	NP	NP	NP	NP	2,16	2,66	-4,71	-2,30
NDFIP2	-1,54	NP	NP	NP	NP	NP	2,16	2,66	-4,71	-2,30
TRIM44	1,69	NP	NP	NP	NP	NP	2,16	2,66	-4,71	-2,30
ERGIC1	-1,60	NP	NP	NP	NP	NP	2,16	2,66	-4,71	-2,30
LMBR1	-1,61	NP	NP	NP	NP	NP	2,16	2,66	-4,71	-2,30
ZBTB1	-1,82	NP	NP	NP	NP	NP	NP	2,66	-4,71	-2,30
POLR1D	-1,53	NP	NP	NP	NP	NP	NP	2,66	-4,71	-2,30
GBA2	1,98	NP	NP	NP	NP	NP	NP	2,66	-4,71	-2,30
SNAPC3	1,61	NP	NP	NP	NP	NP	NP	2,66	-4,71	-2,30
EZH1	1,50	NP	NP	NP	NP	NP	NP	NP	-4,71	-2,30
POLD4	-1,34	NP	NP	NP	NP	NP	NP	NP	-4,71	-2,30
CROCC	1,68	NP	NP	NP	NP	NP	NP	NP	-4,71	-2,30
PHC2	1,06	NP	NP	NP	NP	NP	NP	NP	-4,71	-2,30
RUFY3	1,28	NP	NP	NP	NP	NP	NP	NP	NP	-2,30
WDR73	-1,37	NP	NP	NP	NP	NP	NP	NP	NP	-2,30
BLOC1S1	-1,51	NP	NP	NP	NP	NP	NP	NP	NP	-2,30
UFM1	-1,38	NP	NP	NP	NP	NP	NP	NP	NP	-2,30
C9orf100	1,21	NP	NP	NP	NP	NP	NP	NP	NP	-2,30
RNF7	-1,49	NP	NP	NP	NP	NP	NP	NP	NP	-2,30
AMT	1,77	NP	NP	NP	NP	NP	NP	NP	NP	-2,30

NP= not predicted target accordantly to our criteria; Fold change of miRs and genes was given by the ratio F-BC/NF-BC.
