# Supplementary material for: MicroRNAs Discriminate Familial from Sporadic Non-BRCA1/2 Breast Carcinoma Arising in Patients ≤35 Years
Source: PLoS One. 2014 Jul 9;9(7):e101656. doi: 10.1371/journal.pone.0101656 (PMC4090167; doi:10.1371/journal.pone.0101656)
Supplement: Table S4 — 49 miRNA–mRNA interactions. Interactions presenting significant differences of co-expression profile between F-BC and NF-BC. (PDF) [file pone.0101656.s004.pdf]

Table S4. 49 miRNA–mRNA interactions. Interactions presenting significant differences of co-expression profile between F-BC and NF-BC

GeneSymbol	miR	Absolute co-expression difference	Co-expression F-BC	Co-expression NF-BC	P	Fold-change gene (F-BC/NF-BC)	Fold-chage miR (F-BC/NF-BC)	Predicted/not predicted pair
C20orf12	hsa-miR-660	0,87	-0,5	0,37	0.02	1.64	2.66	predicted
C9orf100	hsa-miR-660	0,76	-0,34	0,43	0.05	1.49	2.66	not predicted
C9orf100	hsa-miR-874	0,75	-0,33	0,42	0.05	1.49	-4.71	not predicted
CA5B	hsa-miR-124	0,84	0,54	-0,3	0.02	-1.93	10.08	predicted
CA5B	hsa-miR-210	0,89	0,72	-0,16	0.02	-1.93	7.32	not predicted
CA5B	hsa-miR-455-3p	0,81	0,43	-0,38	0.03	-1.93	4.29	not predicted
DUSP2	hsa-miR-381	0,79	-0,67	0,12	0.04	2.02	2.47	not predicted
DUSP2	hsa-miR-501-5p	0,95	-0,71	0,24	0.01	2.02	2.16	predicted
EXOC8	hsa-miR-660	0,75	-0,36	0,39	0.05	1.53	2.66	predicted
EZH1	hsa-miR-124	0,78	0,76	-0,02	0.04	1.5	10.08	not predicted
EZH1	hsa-miR-455-3p	0,78	0,76	-0,02	0.04	1.5	4.29	not predicted
EZH1	hsa-miR-486-3p	0,78	0,77	-0,01	0.04	1.5	-4.54	not predicted
EZH1	hsa-miR-660	0,86	-0,66	0,2	0.02	1.5	2.66	not predicted
EZH1	hsa-miR-874	0,86	-0,46	0,41	0.02	1.5	-4.71	predicted
FGD6	hsa-miR-124	0,79	0,51	-0,28	0.04	1.6	10.08	not predicted
FGD6	hsa-miR-874	0,99	-0,44	0,55	0.01	1.6	-4.71	predicted
FKBP4	hsa-miR-660	0,92	0,55	-0,37	0.01	-1.83	2.66	predicted
H2AFZ	hsa-miR-874	0,83	0,47	-0,36	0.03	-1.75	-4.71	predicted
LMBR1	hsa-miR-874	0,77	0,17	-0,6	0.04	-1.61	-4.71	predicted
MAN1A2	hsa-miR-98	0,13	0,2	0,33	0.04	1.58	-2.3	predicted
NAV2	hsa-miR-660	0,87	-0,5	0,37	0.02	2.34	2.66	predicted
NDFIP2	hsa-miR-98	0,3	0,08	-0,22	0.03	-1.54	-2.3	predicted
NDFIP2	hsa-miR-124	0,84	0,76	-0,08	0.03	-1.54	10.08	not predicted
NDFIP2	hsa-miR-210	1,2	0,86	-0,34	0	-1.54	7.32	not predicted
NDFIP2	hsa-miR-381	1,03	0,82	-0,21	0	-1.54	2.47	not predicted
NDFIP2	hsa-miR-486-3p	0,9	0,72	-0,19	0.02	-1.54	-4.54	not predicted
NDFIP2	hsa-miR-501-5p	1,12	0,67	-0,45	0	-1.54	2.16	predicted
NFATC2IP	hsa-miR-124	0,81	0,75	-0,06	0.03	1.71	10.08	not predicted
NFATC2IP	hsa-miR-455-3p	0,81	0,7	-0,11	0.03	1.71	4.29	predicted
NFATC2IP	hsa-miR-486-3p	0,86	0,65	-0,21	0.02	1.71	-4.54	predicted
NFATC2IP	hsa-miR-660	0,76	-0,48	0,28	0.05	1.71	2.66	predicted
NFATC2IP	hsa-miR-874	1,07	-0,7	0,38	0	1.71	-4.71	predicted
NUP35	hsa-miR-381	0,78	-0,72	0,06	0.04	-1.42	2.47	predicted
POLR1D	hsa-miR-210	0,79	0,44	-0,35	0.04	-1.53	7.32	not predicted
PSD4	hsa-miR-874	0,77	-0,52	0,26	0.04	1.69	-4.71	predicted
PSEN2	hsa-miR-660	0,82	-0,59	0,23	0.03	2.19	2.66	predicted
RNF145	hsa-miR-210	0,76	-0,57	0,19	0.05	1.57	7.32	not predicted
RNF145	hsa-miR-501-5p	0,78	-0,46	0,32	0.04	1.57	2.16	predicted
SLC24A3	hsa-miR-210	0,89	-0,5	0,38	0.02	2.07	7.32	not predicted
SLC2A4RG	hsa-miR-381	0,78	0,72	-0,06	0.04	-1.4	2.47	predicted
STAT3	hsa-miR-874	0,89	-0,49	0,4	0.02	1.81	-4.71	predicted
TBRG1	hsa-miR-874	0,75	-0,63	0,12	0.05	1.31	-4.71	predicted
TRIM44	hsa-miR-660	0,75	-0,55	0,19	0.05	1.69	2.66	predicted
TRIM44	hsa-miR-874	0,75	-0,6	0,15	0.05	1.69	-4.71	predicted
VPS13A	hsa-miR-874	0,77	-0,39	0,38	0.04	1.48	-4.71	predicted
ZNF480	hsa-miR-381	0,79	0,59	-0,2	0.04	-1.76	2.47	predicted
ZNF76	hsa-miR-660	0,9	-0,6	0,3	0.01	1.7	2.66	predicted
ZNF76	hsa-miR-874	0,99	-0,6	0,38	0.01	1.7	-4.71	predicted
ZNF92	hsa-miR-98	0,14	-0,04	-0,17	0.01	-1.96	-2.3	predicted

Predicted – miR–mRNA interactions predicted by the miRWalk database; showing different co-expression correlations and different expression profiles; not predicted- miR–mRNA interactions not predicted by the miRWalk database; showing different co-expression correlations and different expression profiles.
