# Supplementary material for: MicroRNAs Discriminate Familial from Sporadic Non-BRCA1/2 Breast Carcinoma Arising in Patients ≤35 Years
Source: PLoS One. 2014 Jul 9;9(7):e101656. doi: 10.1371/journal.pone.0101656 (PMC4090167; doi:10.1371/journal.pone.0101656)
Supplement: Table S5 — miR–mRNA not predicted interactions, presenting inverse correlation. (PDF) [file pone.0101656.s005.pdf]

Table S5: miR–mRNA not predicted interactions, presenting inverse correlation

Non-predicted targets by our criteria	miR pair	Gene fold F-BC/NF-BC	miR fold F-BC/NF-BC	Co-expression difference (P-value)	Other database algorithms (7 mer)
C9orf100*	hsa.miR.874	1.49	-4.70	0.05	Diana, miRanda, Pictar 5
CA5B	hsa.miR.210	-1.93	7.32	0.02	NA
CA5B*	hsa.miR.455.3p	-1.93	4.28	0.03	Diana, miRanda, Pictar 5
EZH1*	hsa.miR.486.3p	1.50	-4.53	0.04	TargetScan, Diana e Pictar
NDFIP2*	hsa.miR.124	-1.54	10.08	0.03	Diana e miRanda
NDFIP2	hsa.miR.210	-1.54	7.32	0	NA
NDFIP2*	hsa.miR.381	-1.54	2.40	0	Diana, miRanda, Pictar 5
POLR1D	hsa.miR.210	-1.52	7.32	0.04	NA

*Five miR–mRNA pairs could be considered as predicted using less stringent criteria.
