# Supplementary material for: MicroRNAs Discriminate Familial from Sporadic Non-BRCA1/2 Breast Carcinoma Arising in Patients ≤35 Years
Source: PLoS One. 2014 Jul 9;9(7):e101656. doi: 10.1371/journal.pone.0101656 (PMC4090167; doi:10.1371/journal.pone.0101656)
Supplement: Table S6 — Biological processes. Categories over represented by the target genes related to 49 miR-mRNA interactions. (PDF) [file pone.0101656.s006.pdf]

Table S6. Biological processes. Categories over represented by the target genes related to 49 miR-mRNA interactions

**Biological processes**	**Genes**	**P**
Apoptosis	*APH1A,APH1B,CD5,DUSP2,EGR2,EGR3, EP400,FKBP4,FKBP8, GLIPR1,HPSE,LIF, PELP1,PHB,PKM, PSEN2,PSENEN, RAC1,STAT3,TP53, VCAN, VPS13A, WT1*	0.00000061
Cell death	*APH1A,APH1B,CD5,DUSP2,EGR2,EGR3, EP400,FKBP4,FKBP8,GLIPR1,HPSE, LIF, PELP1,PHB,PKM, PSEN2,PSENEN, PTGES3,RAC1, SLC16A1,STAT3, TP53,VCAN,VPS13A,WT1*	0.00000657
Proliferation of fibroblasts	*EP400,HPSE,KDM5A,LIF, PTGES3, RAC1, STAT3,TP53, VCAN*	0.00000299

Biological process categories significantly over represented by significant co-expressed genes ($P<0.05$, Fisher´s exact test). Other similar categories were not included to reduce redundancy.
