# Supplementary material for: MicroRNAs Discriminate Familial from Sporadic Non-BRCA1/2 Breast Carcinoma Arising in Patients ≤35 Years
Source: PLoS One. 2014 Jul 9;9(7):e101656. doi: 10.1371/journal.pone.0101656 (PMC4090167; doi:10.1371/journal.pone.0101656)
Supplement: Table S7 — Patient and tumor characteristics of the validation set. (PDF) [file pone.0101656.s007.pdf]

Table S7. Patient and tumor characteristics of the validation set

ID names	Age	ER	PR	HER-2	Subtype	TNM	Grade
F-BC 9	29	POS	POS	NEG	Luminal A	I	NA
F-BC 8	33	POS	NEG	NEG	Luminal A	I	NA
F-BC 5	29	NEG	NEG	NEG	Trip NEG	IIA	GN3 / GH3
F-BC 7	34	POS	POS	NEG	Luminal A	IIB	GN3 / GH2
F-BC 10	28	NEG	NEG	NEG	Trip NEG	IIIB	GN3 / GH3
F-BC 11	32	POS	POS	NEG	Luminal A	IIIA	GH2 / GN2
F-BC 12	29	POS	POS	POS	Luminal B	IIA	GH3 / GN3
NF-BC 27	28	POS	NEG	NEG	Luminal A	IIA	GH3 / GN3
NF-BC 28	30	POS	POS	NEG	Luminal A	IIIA	GH2 / GN2
NF-BC 29	31	POS	POS	POS	Luminal B	IIA	GH3 / GN3
NF-BC 30	29	POS	POS	POS	Luminal B	IIIA	GH3 / GN3
NF-BC 31	29	POS	POS	NEG+	Luminal A	IIIB	GN3
NF-BC 32	34	POS	POS	POS	Luminal B	IIIA	GH2 / GN2
NF-BC 33	32	POS	NEG	NEG	Luminal A	IIB	GH1 / GN2
NF-BC 34	34	POS	POS	POS	Luminal B	IIIA	GH3/ GN3
NF-BC 35	30	POS	POS	POS	Luminal B	IIA	GH3 / GN3

POS: positive; NEG: negative; Trip. NEG: triple negative; GN: nuclear grade; GH: histological grade; ER: estrogen receptor; PR: progesterone receptor; HER-2: growth factor receptor type 2; TNM criteria suggested by WHO (World Health Organization): I (T1N0M0); IIA (T0N1M0, T1N1M0, T2N0M0); IIB (T2N1M0,T3N0M0); IIIA (T0N2M0, T1N2M0, T2N2M0, T3N1M0, T3N2M0); IIIB (T4N0M0,T4N1M0, T4N2M0); IV (every T, N, and M1).
